# Managing Neuropathic Pain in Dogs

**DOI:** 10.3389/fvets.2016.00012

**Published:** 2016-02-22

**Authors:** Sarah A. Moore

**Affiliations:** ^1^Department of Veterinary Clinical Sciences, The Ohio State University, Columbus, OH, USA

**Keywords:** neuropathic pain, spinal cord injury, hyperesthesia, allodynia, dog

## Abstract

Disorders of the somatosensory system such as neuropathic pain are common in people with chronic neurologic and musculoskeletal diseases, yet these conditions remain an underappreciated morbidity in veterinary patients. This is likely because assessment of neuropathic pain in people relies heavily on self-reporting, something our veterinary patients are not able to do. The development of neuropathic pain is a complex phenomenon, and concepts related to it are frequently not addressed in the standard veterinary medical curriculum such that veterinarians may not recognize this as a potential problem in patients. The goals of this review are to discuss basic concepts in the pathophysiology of neuropathic pain, provide definitions for common clinical terms used in association with the condition, and discuss pharmacological treatment options for dogs with neuropathic pain. The development of neuropathic pain involves key mechanisms such as ectopic afferent nerve activity, peripheral sensitization, central sensitization, impaired inhibitory modulation, and pathologic activation of microglia. Treatments aimed at reducing neuropathic pain are targeted at one or more of these mechanisms. Several drugs are commonly used in the veterinary clinical setting to treat neuropathic pain. These include gabapentin, pregabalin, amantadine, and amitriptyline. Proposed mechanisms of action for each drug, and known pharmacokinetic profiles in dogs are discussed. Strong evidence exists in the human literature for the utility of most of these treatments, but clinical veterinary-specific literature is currently limited. Future studies should focus on objective methods to document neuropathic pain and monitor response to therapy in veterinary patients.

## Introduction

Disorders of the somatosensory system such as neuropathic pain affect up to 8% of the general population and up to 90% of people living with chronic spinal cord injury (SCI), yet these conditions remain an underappreciated morbidity in veterinary patients (1, 2). Additionally, veterinarians have historically received limited background in the concepts of neuropathic pain during training, making it more difficult to recognize this potential problem in patients. The goal of this review is to summarize basic concepts in the literature related to somatosensory disturbance and neuropathic pain and to review recent publications related to the diagnosis and management of neuropathic pain in dogs. Terminology related to neuropathic pain is vast, and often misused in both the human and veterinary clinical literature. Definitions of important terms relevant to the discussion of neuropathic pain, as recommended by the International Association for the Study of Pain (IASP), are summarized in Table 1 (3).

**Table 1:** **Summary of terms and definitions relevant to the discussion of neuropathic pain in veterinary patients**.

Term	Definition
Interoceptive stimulus	A sensory stimulus that originates from within the body
Exteroceptive stimulus	A sensory stimulus that originates from outside of the body
Pain	An unpleasant sensory and emotional experience provoked by a damaging or potentially damaging stimulus
Nociceptive pain	Pain caused by a noxious stimulus that is processed by a normally functioning somatosensory system
Neuropathic pain	Pain caused by a disease or lesion causing dysfunction of the somatosensory system
Mixed pain	Condition of coexisting nociceptive and neuropathic pain
Allodynia	Pain provoked by a stimulus that does not normally cause pain
Hyperesthesia	Increased sensitivity to stimulation
Paresthesia	An abnormal sensation (burning, tingling, “skin crawling”) that can be either spontaneous or provoked
Hyperpathia	An abnormally painful reaction to a stimulus
Hypoalgesia	Diminished pain in response to a stimulus that would normally be painful
Analgesia	Absence of pain in response to a stimulus that would normally be painful
Central sensitization	Response of nociceptive neurons within the central nervous system to normally non-painful or sub-threshold sensory stimulus

## The Somatosensory System: An Overview

The somatosensory system serves three major functions. It allows perception and reaction to sensory stimuli originating inside the body (interoceptive), responds to stimuli originating outside the body (exteroceptive), and mediates proprioceptive function (4). The first order neurons in pathways of the somatosensory system reside in the dorsal root ganglia (DRG), cranial sensory ganglia, or the brainstem. The anatomy of DRG neurons has been described as pseudounipolar, meaning that they possess a single-branched axon, which extends both into the periphery to associate with sensory receptors and into the spinal cord to form synapses with second-order neurons in either the dorsal gray matter or brainstem nuclei (4). The general somatic afferent system (GSA) is commonly referred to as the “pain, temperature, touch” system, which is partially a misnomer because pain is not actually a sensory modality but rather a feeling provoked in response to a sensory stimulus. This is the system that transmits information related to thermal, mechanical, and chemical stimuli from peripheral receptors to the somatosensory cortex (5). The general proprioceptive (GP) system is responsible for detecting the movement and position of muscles and joints, encompassing both conscious and unconscious proprioceptive components (5).

## Pain and the General Somatic Afferent System

Pain is classically defined as “an unpleasant feeling provoked by intense or damaging stimuli” (6). The generation of pain in response to tissue injury involves four basic processes: transduction, transmission, modulation, and perception. Transduction involves the conversion of a noxious stimulus to a nociceptive signal at the level of the nociceptor. Transmission is the process by which nociceptive signals are propagated along nerve fibers from the site of original injury to the CNS. Modulation is the mechanism by which nociceptive signals are altered within the CNS through either facilitation or inhibition. Perception is the last and most important part of the “experience” of pain, involving integration of cognitive and emotional responses to the noxious stimulus (7).

In general, pain can be categorized as either nociceptive or neuropathic. *Nociceptive pain* is caused by noxious stimuli which are processed by an otherwise normally functioning somatosensory system – for example pain associated with the production of pro-inflammatory mediators after a broken bone. Nociceptive pain is evolutionarily advantageous by allowing an animal to detect and respond to a potentially damaging stimulus. It can be further classified as somatic (originating from skin, muscles, and joints) or visceral (originating from visceral organs). *Neuropathic pain* is defined as pain caused by a disease or lesion, which leads to damage or dysfunction of the somatosensory system (8). The term *mixed pain* refers to the condition of coexisting nociceptive and neuropathic pain. It is likely that neuropathic and mixed pain are both common but under-recognized phenomena in our veterinary patients with long-standing neurologic, orthopedic, or other diseases.

Neuropathic pain is a maladaptive phenomenon caused by pathologic neuroplasticity, and can become a disease of the neurologic system in its own right by persisting beyond resolution of an inciting cause (9). It is caused by a broad range of conditions affecting any organ or tissue that possesses nerve endings. Specifically, conditions such as peripheral neuropathy, spinal cord disease, chronic musculoskeletal conditions, and brain lesions are commonly reported (1, 2, 10–13). Manifestations of neuropathic pain include both evoked pain (stimulus dependent hypersensitivity) and spontaneous pain. These signs may be either continuous or intermittent in nature. Stimulus-evoked hypersensitivity is often categorized further in the literature in to the two most common types of neuropathic pain recognized and discussed in the clinical setting: allodynia or hyperesthesia (14). The term *Allodynia* originates from the Greek words for other (allo) and pain (odynia), and refers to a condition where a stimulus not typically considered painful and not encoded by nociceptors is perceived to be painful by an individual with somatosensory dysfunction. *Hyperesthesia* refers to a condition of increased sensitivity to a stimulus. The term *Hyperpathia* originates from the Greek meaning “increased suffering” and denotes an exaggerated level of pain in response to a stimulus. Allodynia and hyperesthesia can both be present in conjunction with hyperpathia, and the terms are frequently confused and used incorrectly in both the veterinary and human medical literature.

## Neuropathic Pain Mechanisms

The first step in the establishment of neuropathic pain is development of a lesion somewhere within the somatosensory system. The initiating stimulus is typically associated with nociceptive pain. Under normal circumstances, noxious stimuli diminish as healing occurs, and the experience of nociceptive pain decreases over time. However, intense chronic nociceptive pain can activate mechanisms in both the peripheral and central nervous system (CNS) that lead to the development of a neuropathic pain state. The question of exactly when acute nociceptive pain becomes chronic and deleterious is difficult to answer, although an understanding of the window during which permanent changes to the somatosensory system occur could facilitate better prevention and management of neuropathic pain (15).

The key mechanisms which underlie the development of neuropathic pain conditions include the following: ectopic activity in afferent nerves, peripheral sensitization, central sensitization, impaired inhibitory modulation, and pathologic activation of microglia. These mechanisms can occur across as spectrum of pathologic processes. Additionally, within a single patient, multiple neuropathic pain mechanisms may be present, and different mechanisms can lead to the same clinical signs. Importantly, in people, efficacy of a particular drug to treat neuropathic pain is not generally dependent on the underlying cause but rather the type of neuropathic pain syndrome displayed (16). This highlights the complexity of neuropathic pain development and maintenance and underscores the need to consider treatment plans for each patient on an individual basis (14, 17). Mechanisms that underlie the development of neuropathic pain are discussed in detail below.

### Ectopic Afferent Nerve Activity

Ectopic afferent activity refers to injury-induced hyperexcitability, which generates aberrant action potentials in primary afferent neurons or in other sites along the nociceptive pathway (2). People with ectopic afferent activity report the sensation of ongoing spontaneous pain, or paroxysmal shooting pain in the absence of an external stimulus (14). Ectopic afferent activity may originate from damaged nerve fibers or uninjured adjacent fibers, which become activated as a result of non-synaptic signal transmission (7, 9). This ectopic activity is caused by sub-threshold oscillations in resting membrane potential, which result in bursts of rhythmic nerve depolarization in the absence of an appropriate stimulus. Inflammatory processes ensue, leading to a state of peripheral sensitization.

### Peripheral Sensitization

Peripheral sensitization is characterized by a reduced threshold and increased intrinsic excitability of peripheral nociceptor terminals. In most cases, peripheral sensitization will resolve as healing occurs. However, it may persist in scenarios of continued disease or injury, leading to physical alterations in the functions of primary afferent neurons (18). Sodium channels are considered the primary player in this phenomenon, where increased numbers of heterotopic sodium channels such as Nav1.8, Nav1.7, and Nav1.3 lower stimulus threshold and result in neuropathic pain (19–22).

### Central Sensitization

Central sensitization refers a phenomenon by which nociceptive input is amplified within the CNS in patients with neuropathic pain. Central sensitization occurs when dorsal horn synaptic plasticity results in persistent abnormal nociceptive processing. After injury, repeated discharges from GSA neurons leads to the release of excitatory molecules within the dorsal horn of the spinal cord. This causes postsynaptic effects on second-order nociceptive neurons such as increased expression of voltage-gaited sodium channels and alterations in AMPA and NMDA receptors (23, 24). These changes enable low-threshold mechanosensitive fibers to aberrantly activate second-order nociceptive neurons. A potential consequence is that normally innocuous tactile stimuli such as light touch can cause pain.

### Pathologic Activation of Microglia

Neuroinflammatory mechanisms, including microglial activation, are also implicated in the induction and maintenance of a chronic neuropathic pain state (25). As a result of injury, microglia within the CNS become pathologically activated and respond by releasing inflammatory mediators such as ATP and CCL2 (6). These, in turn, lead to activation of astrocytes and enhanced production of other inflammatory mediators such as TNF, IL-1β, and IL-6. These pro-inflammatory cytokines have been shown to play a vital role in sensitization in rodent models of neuropathic pain by inducing changes in central modulation of painful stimuli and hyperexcitability of nociceptive neurons (26, 27).

## Evaluation of Neuropathic Pain in the Veterinary Setting

Chronic somatosensory dysfunction, primarily in the form of neuropathic pain, is common in people living with chronic SCI and other diseases (1, 2). Neuropathic pain is also a well-described phenomenon in rodent models of traumatic brain injury and stroke, in chronic musculoskeletal disease, neuropathy, and other illnesses (10–13). In rodents, the presence of neuropathic pain can be assessed using objective laboratory techniques such as quantitative sensory testing (QST), which are discussed in greater detail below. The presence of neuropathic pain in veterinary patients with similar conditions has been explored only in a limited fashion. This is likely because neuropathic pain in people is typically assessed using bedside examination, where patients self-report their pain (14). Dogs, of course, are unable to self-report. Additionally, until recently, QST techniques have been viewed more as laboratory tools instead of clinical assessments. As such, they have not been employed commonly in the veterinary clinical setting.

### History Taking and the Clinical Examination

Veterinary patients with neuropathic pain may display certain clinical signs either visible on clinical examination or reported by owners during history taking. Pet owners may not realize their pet is displaying signs of pain, so veterinarians should question carefully for potential signs in patients at risk. Obvious manifestations may include altered reaction to touch, vocalization in the absence of an overt painful stimulus, phantom scratching, excessive licking or self-mutilation, and persistent lameness/diminished weight-bearing on a limb. More subtle signs may include decreased general activity level, reluctance to climbing or descending stairs, diminished jumping behavior, difficulty rising from a seating position, trouble getting into and out of the car, changes in body posture, and altered demeanor or appetite (28, 29).

### Quantitative Sensory Testing

“Sensory testing” in dogs has been historically limited to the context of severe SCI, with results inferring prognosis for neurologic recovery (30). This testing involves subjective evaluation of the presence or absence of a behavioral response to a strong nociceptive stimulus such as pinching the toes – interpreted as the “presence” or “absence” of intact nociceptive pathways. At best, response can be evaluated as normal, diminished, or absent. While an important clinical prognostic indicator in SCI, this subjective evaluation provides no ability to assess the presence of neuropathic pain, or objectively quantify its severity.

Objective methods to evaluate abnormalities in sensory function, such as QST, have been developed for use in rodent models of SCI and more recently in the human clinical setting (31–33). These techniques are useful in diagnosing neuropathic pain and in assessing response to treatment interventions aimed its severity. Methods of QST produce an objective value called sensory threshold (ST) for an array of stimuli such as pin-prick, light touch, vibration, and temperature. In patients with diseases that produce diminished sensation, ST will be higher than normal (hypoalgesia). In patients with diseases that produce neuropathic pain, ST will be lower than normal (hyperesthesia). QST is an important part of outcome assessment in translational laboratory animal models of acute and chronic pain; however, these techniques have not routinely been used in the veterinary clinical setting.

Techniques for QST can also be valuable in assessing dogs with various medical conditions (34–36). Procedures for the use of devices for mechanical QST using, such as the electronic von Frey anesthesiometer (VFA), have been weight-bearing in client-owned dogs (34–39). Mechanical QST provides an objective evaluation of the amount of mechanical pressure that must be applied to the patient (in dogs, typically the dorsal surface of the paw with the patient positioned in lateral recumbancy) to produce a behavioral response indicating conscious perception of the stimulus. The pressure necessary to produce this response is recorded as the “ST” for the patient. Using this technique, hyperesthesia is manifest as a reduced ST. These techniques have potential utility in diagnosing neuropathic pain in dogs, and monitoring response to therapies; however, they may be influenced by the technique of the investigator and the emotional state of the patient, indicating that further work is needed to refine this technique before it can be adopted for routine clinical use (39). In rodent laboratory studies using similar techniques, a “gentling period” of several days is typically suggested to allow the animal to acclimate to the testing device and environment prior to initiating testing (33). This recommendation stems from the knowledge that anxiety can affect the character of behavioral responses observed during QST. Obviously, in veterinary patients with spontaneously occurring diseases this approach is not feasible. For dogs, a 10–15-min acclimation period to the testing room has been suggested in conjunction with maintaining a quiet comfortable space with limited traffic and distractions (34). Testing using the VFA technique involves assessing ST in each limb via five stimuli applied at least 1 min apart to prevent ST decay between tests (35). Thus, the technique requires approximately 30 min per dog per testing session to accomplish if all four limbs are evaluated.

Williams et al. have also recently reported the feasibility of thermal QST in normal dogs and those with pain associated with osteoarthritis (40). This technique assesses thermal threshold latency (TTL) of the ventral surface of the paw with the dog in a standing position. The limb is positioned on a glass plate and a light source applied below the glass centered on the pad of the third digit. The stimulus is applied for a maximum of 30 s and terminated automatically when the dog lifts its foot or moves off the glass plate. The time between stimulus application and automatic termination of the stimulus is recorded as the TTL.

## Managing Neuropathic Pain

The first, and perhaps most important step, in the management of neuropathic pain is identification and treatment (whenever possible) of the underlying disease affecting the somatosensory system. There are many conditions common in veterinary patients, which are likely to result in neuropathic pain based on what we know from analogous human conditions. Examples include Chiari-like malformation/syringomyelia (CM/SM), radiculopathy caused by chronic cervical or lumbosacral disc disease, diabetic or other polyneuropathy, SCI caused by intervertebral disc extrusion, chronic osteoarthritis, and stroke (1, 2, 10–13).

Many of the conditions associated with neuropathic pain may also have a clinical component of nociceptive pain. Strategies for managing nociceptive pain have been reviewed extensively in the recent veterinary literature and will not be discussed here (41, 42). Management of neuropathic pain in the veterinary setting presents challenges, as apparent in CM/SM, where the clinical manifestations of neuropathic pain may be refractory to treatment and often progress over time (43, 44). Treatments can be targeted at any or all of the five mechanisms underlying neuropathic pain, which were described above. A recent meta-analysis of the human neuropathic pain literature makes strong recommendations for the use of drugs such as gabapentin, pregabalin, and tricyclic antidepressants (TCA), while weaker recommendations are made for tramadol and strong opioids such as morphine (related to both efficacy and side effects) and topical lidocaine or capsaicin (45). The most common drugs used specifically for neuropathic pain the veterinary setting are gabapentin and pregabalin, with TCA occasionally referenced (46, 47). A summary of dosing recommendations for medications used to manage neuropathic pain in dogs can be found in Table 2.

**Table 2:** **Common medications used for the management of neuropathic pain in dogs**.

Drug	Dosage (mg/kg)	Frequency (h)	Reference
Gabapentin	10–20	Q8	(48, 49)
Pregabalin	4	Q12	(50)
Amitriptyline	3–4	Q12	(51)
Amantadine	3–5	Q12–24	(42, 52)

### Gabapentin

Gabapentin was originally marketed as an anticonvulsant medication but was subsequently found to have beneficially effects in the management of neuropathic pain (53, 54). It is currently approved by the FDA for use in postherpes neuralgia. The extended release formulation, gabapentin enacarbil, is approved for use in restless leg syndrome (RLS) (42). Gabapentin is a structural analog of GABA. It appears to decrease central sensitization by inhibiting presynaptic calcium channels in the dorsal horn (55). Gabapentin may also have efficacy related to sodium channel blockade and elimination of ectopic nerve activity (55).

The current evidence for gabapentin as an analgesic medication in dogs is low; however, it is commonly used in the clinical setting. Several studies have examined the efficacy of gabapentin as an adjunctive analgesic after surgical procedures such as forelimb amputation, mastectomy, and hemilaminectomy (56–58). Wagner et al. (56) found no difference between gabapentin and placebo to reduce the need for postoperative opioids when dosed at 10 mg/kg Q12 h in patients undergoing forelimb amputation. Aghighi et al. (57) found no difference in gabapentin compared to placebo in management of postoperative pain after intervertebral disc extrusion. Crociolli et al. (58) found that perioperative administration of gabapentin at 10 mg/kg immediately prior to surgery and Q12 h for 3 days following mastectomy significantly decreased postoperative opioid requirements. The fact that all three studies used a dosing frequency of Q12 h may explain the mixed results, as previous pharmacokinetic studies have suggested appropriate dosing is 10–20 mg/kg every 8 h in dogs (48, 49). Additionally, gabapentin was evaluated acutely after injury in all three studies. Because neuropathic pain is a chronic phenomenon, it is likely that gabapentin’s efficacy to manage nociceptive pain was the only thing truly being evaluated. Several case series discuss the role of gabapentin in the management of chronic neuropathic pain associated with CM/SM, but there are no controlled studies in the veterinary literature (59–61). Additionally, a recent study compared gabapentin to topiramate as an add-on to carprofen in the management of CM/SM (59). This study reported no improvement in pain scores assigned via visual analog scale; however, improvement in quality of life scores for dogs receiving gabapentin was observed. While it is likely that gabapentin has efficacy against neuropathic pain based on the human literature and on anecdotal evidence, controlled studies will be required to understand the role of gabapentin to manage neuropathic pain in veterinary patients.

### Pregabalin

Pregabalin is structurally similar to gabapentin but has higher oral bioavailability and a longer half-life (42, 50). In a single-dose pharmacokinetic study performed in normal dogs, a dose of 4 mg/kg produced plasma drug concentrations within the predicted therapeutic range extrapolated from the human literature (50). Elimination half-life of the drug in that study supported Q12-h dosing. There are no controlled studies evaluating the efficacy of pregabalin to treat neuropathic pain in veterinary patients, although a case series of dogs with CM/SM reports its use (47). The current cost of pregabalin often prevents its use as a first line drug for veterinary patients.

### Tricyclic Antidepressants

The effects of neuromodulatory drugs such as TCA in managing neuropathic pain are distinct from their antidepressant effects (62). TCAs including amitriptyline are considered a first-line therapy for neuropathic pain in humans, with demonstrated efficacy in diseases such as postherpetic neuralgia, poststroke pain, and chronic low back pain (63). TCAs exert analgesic effects in several ways, including inhibiting reuptake of serotonin and norepinephrine, antagonism of voltage-gaited sodium channels, and antagonism of NMDA receptors. There are no clinical trials, or experimental studies evaluating the use of TCAs for neuropathic pain in dogs, but a case for their utility can be made based on the human literature. A small case series describes the management of neuropathic pain in three dogs with mixed results using amitryptiline alone or in conjunction with other analgesics (46). A recent pharmacokinetic study of amitryptiline in dogs suggests that dosing at 3–4 mg/kg every 12 h will reach target drug concentrations extrapolated from people (51).

### Amantadine

Amantadine is an NDMA antagonist that has shown mixed results in the treatment of neuropathic pain in humans (64). Amantadine may decrease central sensitization, decreases opioid tolerance in some patients, and is suggested to enhance the effects of NSAIDS, gabapentin, and opioids (65). Dosing at 3–5 mg/kg Q24 h is typically suggested throughout the veterinary literature, although a recent pharmacokinetic study of single-dose amantadine in greyhounds suggests that Q12-h dosing might be more rational (42, 52, 65, 66). A single case report describes the use of amantadine specifically for neuropathic pain in a dog, and no studies evaluating the analgesic effects of amantadine specifically to treat neuropathic pain are available in dogs (67). However, a randomized, placebo-controlled trial evaluated the use of amantadine in conjunction with meloxicam in dogs with osteoarthritis and showed significant improvement in pain scores for dogs receiving amantadine compared to placebo (65).

### Opioids

The role of opioids in the management of people with neuropathic pain is both controversial and complex. A recent meta-analysis found moderate evidence for their use to decrease the intensity of neuropathic pain with intermediate-term treatment, but also cited significant bias in currently available studies, and the need for prospective studies focusing on adverse effects (68). Recent studies also indicate that the use of opioids in some patients may contribute to a condition called opioid-induced hyperalgesia (69). Opioid-induced hyperalgesia is characterized by a heightened state of pain sensation characterized by both a lowered pain threshold and a decrease in tolerance for pain (70). Patients with opioid-induced hyperalgesia report increases in sensitivity to pain and higher pain scores as opioid doses are increased. The exact mechanism by which this occurs is not completely understood, but opioid effects on NMDA and μ receptors are likely involved (71). In the context of postoperative pain, extensive use of opioids prior to a procedure has been associated with a more painful and prolonged recovery (72). Drugs considered strong opioid agonists, such as oxycodone and morphine, show moderate efficacy in the management of neuropathic pain but have significant side effects such as constipation of other gastrointestinal disturbances with long-term use. Tramadol is considered a weak opioid agonist, but may also exert effects similar to TCAs by inhibiting reuptake of serotonin and noradrenaline to aid in the management of neuropathic pain (73).

### Cannabinoids

Of recent interest in the management of neuropathic pain are the cannabanoids. Cannabinoids are a family of chemicals derived from the cannabis (marijuana) plant. Many pharmacologically active cannabinoids have been identified but the two with the strongest evidence for analgesic effects are tetrahydrocannabinol (THC), which also has psychoactive effects, and cannabidiol (CBD). Cannabinoids exert their effects within the body via two well-studied receptors: CB_1_ and CB_2_. CB_1_ receptors are present in high numbers in the brain and spinal cord, as well as visceral organs and adipose tissue. Activation of CB_1_ receptors leads to inhibited release of acetylcholine, dopamine, and glutamate. CB_1_ also modulates opioid, NMDA, and GABA receptors (74, 75). Most known adverse effects associated with cannabinoids are associated with central CB_1_ receptor effects (76). CB_2_ receptors are found in the highest concentrations on hematopoietic and immune cells, including microglial cells (75, 76). In normal health, CB_2_ receptors are expressed only at low levels in the CNS but are rapidly upregulated in both neurons and microglia after injury or inflammation (76). Several peripherally restricted CB_1_ or CB_2_ receptor agonists have been developed to circumvent the centrally meditated side effects of cannabinoids. These drugs have been evaluated for the treatment of inflammation and neuropathic pain. Studies have shown mixed results, a summary of which can be found in a recent review of the human literature regarding cannabinoids in pain management (76). Additionally, a recent meta-analysis made weak recommendations against the use of cannabinoids for management of neuropathic pain (45). While medical marijuana or cannabis-based products have not historically been a topic for discussion in veterinary medicine, the use of cannabinoids deserves mention here for two reasons. First, future studies may support the use of novel restricted CB_1_ or CB_2_ receptor agonists in veterinary patients. Additionally, a commercially available hemp-based supplement has been recently marketed for use in dogs and is available online without a prescription (Canna-Pet^®^). This product has not been evaluated by the FDA for cannabinoid content, or efficacy. No quantitative information regarding CBD or other cannabinoid content is listed on the website for this product. Additionally, the company declined to provide quantitative information on CBD content of their products when requested by the author specifically for inclusion in this review.

## Non-Pharmacologic Management

Several non-pharmacologic techniques for managing neuropathic pain are suggested throughout the veterinary literature. Examples include acupuncture, medical massage, cold or heat therapy, shock wave therapy, platelet-rich plasma, and dietary supplements (29). While veterinary-specific literature is limited with respect to each of these modalities in the management of neuropathic pain, a recent review article summarizes the concepts behind utility of these techniques for pain management in dogs and cats (77).

## Summary

Neuropathic pain is likely an under-recognized condition in veterinary patients with an assortment of neuromusculoskeletal diseases. Specific literature related to diagnosis and treatment of neuropathic pain in dogs is limited, although information related to mechanisms and treatment options can be extrapolated from the human literature. Drugs specifically targeted at reducing neuropathic pain, such as gabapentin and pregabalin, will require controlled prospective studies evaluating their efficacy to reduce clinical signs compatible with neuropathic pain such as hyperesthesia and allodynia. Veterinary clinicians should be aware of neuropathic pain as a potential entity in patients and should consider it when developing a multimodal approach to pain management.

## Author Contributions

SM drafted and revised the manuscript.

## Conflict of Interest Statement

The author declares that the research was conducted in the absence of any commercial or financial relationships that could be construed as a potential conflict of interest.
